# Primary hepatic carcinoid tumor

**DOI:** 10.1186/1477-7819-9-151

**Published:** 2011-11-19

**Authors:** Jinbo Gao, Zhijian Hu, Junwei Wu, Lishan Bai, Xinqun Chai

**Affiliations:** 1 Department of Hepatobiliary Surgery, Union Hospital, Tongji Medical College, Huazhong University of Science and Technology, 1277 Jiefang Avenue, Wuhan 430022, China

**Keywords:** carcinoid tumor, liver, surgery

## Abstract

Primary hepatic carcinoid tumor is rare and poses a challenge for diagnosis and management. We presented a case of primary hepatic carcinoid tumor in a 53-year-old female with a complaint of right upper abdominal pain. Computer tomography scans revealed a hypervascular mass in segment 4 of the liver. An ultrasonography-guided biopsy showed a carcinoid tumor. No other lesions were found by the radiological investigations. Surgery resection was performed and histopathological examination revealed a primary hepatic carcinoid tumor. Three years later, recurrence was found and transcatheter arterial chemoembolization was performed. After transcatheter arterial chemoembolization, the patient has been free of symptom and had no radiological disease progression for over 6 months. Surgical resection combination with transcatheter arterial chemoembolization is effective to offer excellent palliation.

## Background

Carcinoid tumors are neoplasms that originate from the cells of neuroendocrine system, and were first characterized by Oberndorfer in 1907. They are usually low-grade malignant tumors that may cause the carcinoid syndrome by secretion of serotonin and other vasoactive hormones. Most of them occur within the gastrointestinal tract, primarily in the appendix and the terminal ileum [1,2]. Primary hepatic carcinoid tumors (PHCT) are very rare. Here, we report a case of PHCT, and describe the clinical features and treatment of this case.

## Case report

A 53-year-old female presented with right upper quadrant abdominal pain and was admitted to our hospital in May 2007. The patient had no history of jaundice, vomiting, flushing or diarrhea. Her past medical history included hypertension and a cholecystectomy due to gallstones 10 years previously. Physical examination showed no abnormality except for the presence of a scar in the right upper quadrant of abdomen. Laboratory investigations, including liver function test, renal function test, blood routine test and tumor markers (AFP, CEA, CA19-9, CA242, CA125 and CA15-3), were within normal limits. Serum hepatitis B surface antigen and hepatitis C antibody were negative.

**Figure 1:**
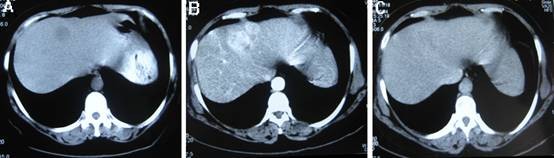
**Computed tomography (CT) of the liver**. A. Noncontrast CT image shows a low density mass in segment 4 of liver. B, C. Dynamic CT scans show enhanced of the nodule in the arterial phase and early washout in the portal phase.

**Figure 2:**
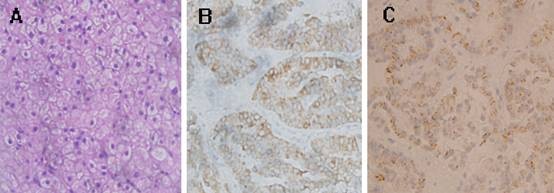
**Histological findings of the liver tumor**. A. Histological examination showed that the tumor cells were round or columnar cells with round nuclei and pale eosinophilic cytoplasm (HE, ×400). B. Immunohistochemistry revealed the tumor cells were positive for synaptophysin (×400). C. Immunohistochemistry revealed the tumor cells were positive for chromorgranin A. (×400).

Computer tomography (CT) revealed a low density nodule in segment 4 of liver. Dynamic CT scans showed enhanced of the nodule in the arterial phase and early washout in the portal phase (Figure 1). Liver ultrasound confirmed the presence of the hypervascular mass. No enlarged lymph nodes were found by CT or ultrasonic. Based on the imaging finding, hepatocelluar carcinoma was highly suspected. A fine-needle aspiration biopsy was performed under ultrasonographic guidance for definite diagnosis. The histological examination of the biopsy specimens suggested the tumor was a carcinoid tumor. The neoplastic cells were round or columnar cells with round nuclei and pale eosinophilic cytoplasm. Immunohistochemistry was positive for pancytokeratin AE1/AE3, synaptophysin and chromorgranin A. There was negative staining for AFP, hepatocyte, CK-7, CK-20 and villin (Figure 2). To rule out the possibility of metastatic carcinoid tumor in the liver, more investigations were performed, including upper and lower gastrointestinal series, a small bowel series and chest and abdominal CT scans. All these investigations were negative.

In May 2007, left hepatectomy was carried out. No asites was noted and the liver was not cirrhotic. Extensive abdominal exploration showed no any other site of primary tumor. The operation was performed successfully and the postoperative recovery was uneventful. The resected liver specimen contained a 4.0 cm tumor. Histological and immunohistochemistry of the tumor confirmed the findings of the biopsy and the resection line was free from tumor invasion.

At three years after operation, MRI showed multiple lesions of the liver. No other lesions were found by the radiological investigations which include upper gastrointestinal endoscopy, a small bowel series, colonoscopy, chest CT and abdominal CT scans. The recurrence of carcinoid tumor was highly suspected. Due to the multiple lesion of the liver, resection was not feasible and 4 times of transcatheter arterial chemoembolization (TACE, consisting of 0.75 g 5-Fu, 20 mg adriamycin, 8 ml lipiodol and spongel) was performed. Six months after the last TACE, the patient was free of symptom and had no radiological disease progression.

## Discussion

Carcinoid tumors are defined as neuroendocrine tumor. The origin of primary hepatic carcinoid tumors is not well known. They may arise from scatter neuroendocrine cells in the intrahepatic biliary epithelium. It is also hypothesized that chronic inflammation in biliary system may initiate intestinal metaplasia, which predisposes to the development of neuroendocrine tumors. Another possibility is that they originate from ectopic pancreatic or adrenal tissues found within the liver [3].

PHCT are very rare. There are only about 94 cases reported in the current literature [4]. PHCT occur mainly in middle age and is slightly more frequent in females (58.5%). The symptoms of PHCT are non-specific, such as abdominal pain, abdominal mass, fatigue, and weight loss. More than 10% cases are asymptomatic. Only a small percent of patients present the typical carcinoid syndrome (skin flushing, abdominal pain and diarrhea) [4]. The case we presented was a 53-year-old female suffered with right upper quadrant abdominal pain.

Ultrasound is an inexpensive and easily available imaging test for evaluation of liver lesions. Solid masses with cystic areas and hyperechoic or mixed pattern are common findings of PHCT detected by ultrasonic. Contrast-enhanced US showed a rapid and dense enhancement without parenchymal stain [5,6]. Concerning CT findings in PHCT, in most cases noncontrast images show low-density masses, and some have cystic component. Dynamic contrast CT shows enhanced masses in the early phase and low density masses in the late phase [7]. On MRI PHCT usually present with low intensity in T1-weighted images and high intensity on T2-weighted images. Angiography revealed hypervascular masses with or without staining in most cases [8]. In radiological examination, PHCT are detected as hypervascular solid masses with or without cystic areas.

The diagnosis of hepatic carcinoid tumor is mainly based on histological and immunohistochemistry examination. In our case, histological examination of the biopsy specimen and the resected specimen revealed that the tumor cells were round or columnar cells with round nuclei and pale eosinophilic cytoplasm. In immunohistochemistry these cells showed positive for the neuroendocrine markers synaptophysin and chromorgranin A. The differentiation between primary and secondary hepatic carcinoid tumor is impossible identified by histology alone. Because liver is a common site for carcinoid metastasis, the diagnosis of PHCT requires intensive preoperative imaging, operative inspection and long-term follow-up. In our case, the patient was followed up for about 4 years after resection. Any other extrahepatic lesion was not found radiologically during follow-up, except for recurrence in the liver three years later after resection. Therefore, we diagnosed this tumor as a PHCT.

Surgical resection is the preferred treatment for PHCT and has provided favorable outcomes. One study showed that postoperative 1-, 5-, and 10-year survival rates were 88%, 80% and 68%, respectively [9]. Liver transplantation has been reported in a small number of unresectable patients. Studies showed that liver transplantation is a feasible and affordable option in patients with unresectable PHCT that gives good long-term results for disease-free survival [10]. Transcatheter arterial chemoembolization has been reported to achieve good palliation in some unresectable patients [11-13]. TACE was also effective for the recurring tumor [14]. TACE is recommended for cases with unresectable and/or recurrence tumors, but the long-term survival is not usually good enough. The recurrence rate of PHCT at 5 years after resection was as high as 26% [15]. In our case, the recurrence was shown three years after operation, and then TACE was performed. Although the recurrence of PHCT was found, surgical resection combination with TACE seemed effective to offer excellent palliation in the current case.

## Conclusion

In summary, primary hepatic carcinoid tumor is a rare tumor. The diagnosis of primary hepatic carcinoid tumor is base on the histological examination and the exclusion of metastasis disease. Surgical resection is the preferred treatment. Transcatheter arterial chemoembolization is effective to offer excellent palliation for the multiple recurrence lesions.

## Abbreviations

PHCT: Primary hepatic carcinoid tumors; CT: Computer tomography; TACE: transcatheter arterial chemoembolization.

## Consent

Written informed consent was obtained from the patient for publication of this case report and any accompanying images. A copy of the written consent is available for review by the Editor-in-Chief of this journal.

## Competing interests

The authors declare that they have no competing interests.

## Authors' contributions

GJ and CX wrote the manuscript. HZ, WJ and BL collected and analyzed the clinical data. GJ and HZ performed the histological examination. All authors read and approved the final manuscript.
